# Screening of functional and positional candidate genes in families with common variable immunodeficiency

**DOI:** 10.1186/1471-2172-9-3

**Published:** 2008-02-07

**Authors:** Ulrich Salzer, Carla Neumann, Jens Thiel, Cristina Woellner, Qiang Pan-Hammarström, Vassilis Lougaris, Tina Hagena, Johannes Jung, Jennifer Birmelin, Likun Du, Ayse Metin, David A Webster, Alessandro Plebani, Viviana Moschese, Lennart Hammarström, Alejandro A Schäffer, Bodo Grimbacher

**Affiliations:** 1 Division of Rheumatology and Clinical Immunology, Medical School, University Hospital Freiburg, Hugstetterstr. 55, 79106 Freiburg, Germany; 2 Division of Clinical Immunology, Karolinska University Hospital Huddinge, SE-14186 Stockholm, Sweden; 3 Department of Pediatrics and Institute of Medicine "Angelo Nocivelli", University of Brescia, Brescia, Italy; 4 Dept. of Immunology and Molecular Pathology, Royal Free Hospital, University College London, Pond Street, London NW3-2QG, UK; 5 Division of Pediatric Immunology, SB Ankara Diskapi Children's Hospital, Ankara, Turkey; 6 Policlinico Tor Vergata, University of Tor Vergata, Viale Oxford, 81, Rome, Italy; 7 National Center for Biotechnology Information, NIH, DHHS, Bethesda, Maryland, USA

## Abstract

**Background:**

Common variable immunodeficiency (CVID) comprises a heterogeneous group of primary antibody deficiencies with complex clinical and immunological phenotypes. The recent discovery that some CVID patients show monogenic defects in the genes encoding ICOS, TACI or CD19 prompted us to investigate several functional candidate genes in individuals with CVID.

**Results:**

The exonic, protein coding regions of the genes encoding: APRIL, BCMA, IL10, IL10Rα, IL10Rβ, IL21, IL21R, and CCL18, were analyzed primarily in familial CVID cases, who showed evidence of genetic linkage to the respective candidate gene loci and CVID families with a recessive pattern of inheritance. Two novel SNPs were identified in exon 5 and exon 8 of the IL21R gene, which segregated with the disease phenotype in one CVID family. Eleven additional SNPs in the genes encoding BCMA, APRIL, IL10, IL10Rα, IL21 and IL21R were observed at similar frequencies as in healthy donors.

**Conclusion:**

We were unable to identify obvious disease causing mutations in the protein coding regions of the analyzed genes in the studied cohort.

## Background

Common variable immunodeficiency (CVID) is the most prevalent symptomatic primary immunodeficiency in adults. CVID clinically presents with a history of recurrent infections at mucous membranes which is the consequence of a marked hypogammaglobulinemia [1]. The clinical course of CVID is complicated by a plethora of systemic immunopathology including autoimmunity, lymphoproliferation, malignancy and sarcoid-like granulomas.

CVID affects males and females equally and usually manifests in the second or third decade of life. A second peak of onset exists in childhood between the ages of 2 and 5 years [2,3]. The prevalence of CVID in the Western hemisphere is estimated to be approximately 1: 25.000 [1,4].

The majority of CVID cases are sporadic, while approximately 10 to 20% of CVID cases show at least one additional family member affected either by CVID or selective IgA deficiency (sIgAD) [5,6]. Most multiplex CVID families show an autosomal dominant mode of inheritance but about 20% present with a recessive trait. CVID and sIgAD deficiency may concur in a single kindred and sIgAD may show progression to CVID in some affected individuals over time [6-8].

Early association and more recent genetic linkage studies have revealed several putative susceptibility loci for CVID/sIgAD. While most of these studies combined families with sIgAD and CVID, Vořechovský et al analyzed sIgA-deficient families separately and identified a susceptibility locus within the HLA region on chromosome 6p termed "*IGAD1*" [9]. In 2003, the same group reported that certain *HLA-DQ/DR *haplotypes and not other putative candidate genes within the *IGAD1 *region confer the protection from or susceptibility to sIgAD and CVID [10]. The same study suggested that the best prospects for non-MHC susceptibility loci could be found on chromosomes 4p, 12p and 14q, but none of these three loci reached statistical significance.

We were recently able to demonstrate genetic linkage to chromosome 16q for autosomal dominant CVID in this cohort of families [9,10] by reanalysis of existing data restricted to the families with at least one case of CVID, in combination with additional fine-mapping [11]. Furthermore, genome-wide linkage analysis in a large multiplex CVID family revealed linkage to a locus on chromosomes 4q. This locus on 4q was replicated (requiring a lower standard of evidence than initial linkage) in a large cohort of small families [12].

Thus, there is considerable genetic heterogeneity in CVID, which mirrors the variable clinical presentation of the disease. Despite these conundrums, the discovery of genetic defects in ICOS [13,14], TACI/*TNFRSF13B *[15,16] and CD19 [17] provides proof that defects in single genes are associated with a CVID phenotype and underscores the value of candidate gene approaches in CVID. Furthermore, a considerable number of patients carrying mutations in the above mentioned genes were derived from familial cases showing recessive patterns of inheritance and sometimes involving consanguinity. The only CVID gene for which heterozygous mutations have been found in sporadic patients is TACI/*TNFRSF13B *[15,18].

**Figure 1:**
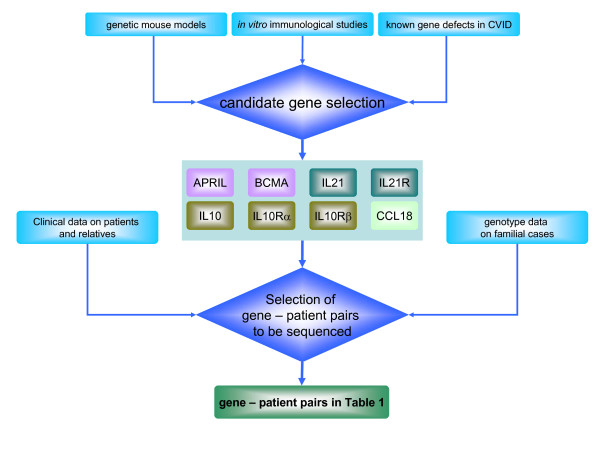
Selection of patients and candidate genes.

We therefore, focused in our candidate gene approach primarily on familial CVID cases and multiplex CVID/sIgAD families, which showed evidence for genetic linkage to the respective candidate gene loci based on available genotype data [9,10]. We concentrated on cytokine/receptor pairs, which showed involvement in terminal B cell differentiation, preferential expression in germinal centers and involvement in class switch recombination. More importantly, the available knockout mouse models of the studied genes present with phenotypes that partially resemble aspects of human common variable immunodeficiency. A flow chart summarizing the study design and selection criteria for patients and candidate genes is provided in Figure 1.

In BCMA/*TNFRSF17*-deficient mice, the generation of long lived plasma cells in the bone marrow is severely impaired [19], which mirrors pathology observed in individuals with CVID [20]. APRIL/*TNFSF13 *is an important factor that drives both human and murine B cells to induce Ig heavy chain class switch to IgA and IgG [21]. Moreover, *TNFSF13*-deficient mice show a selective deficiency in IgA [22].

Interleukin 10 (IL10) is an important immunoregulatory cytokine, which supports terminal B cell differentiation and has potent anti-inflammatory effects. Furthermore, IL10 production by T cells is deficient in human ICOS deficiency [14], X-linked lymphoproliferative disease [23] and in subsets of CVID patients [24], further underscoring the potential involvement of IL10 in the pathogenesis of hypogammaglobulinemia. Finally, IL10-deficient mice develop inflammatory bowel disease, a condition reminiscent of what can be observed in patients with CVID [3].

Signaling via IL21 and the IL21 receptor can induce apoptosis in B cells [25]. However, the interaction is also crucial for terminal B cell differentiation [26,27]. The ability of IL21 to drive plasma cell differentiation makes this gene an attractive candidate gene for human CVID.

Chemokine (C-C motif) ligand 18 (CCL18) is a potent chemoattractant for T cells and B cells, highly expressed in germinal centers of secondary lymphoid organs by dendritic cells [28].

## Results

**Figure 2:**
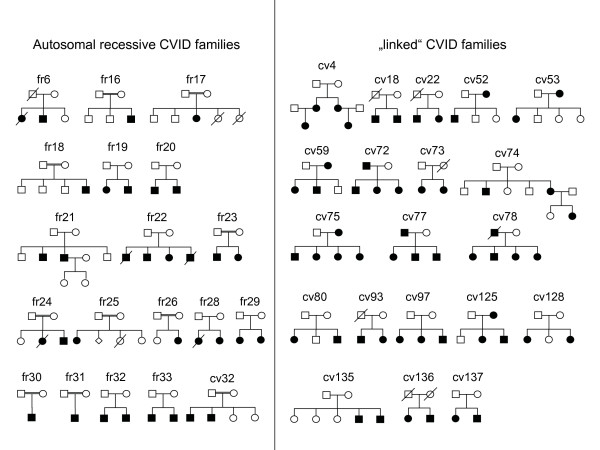
**Pedigrees of CVID families**. Left column: pedigrees of analyzed autosomal recessive CVID families; right column: pedigrees of selected CVID families based on positional reasoning with respect to candidate loci; symbols: circles, female; squares, male; filled symbols, affected individual; slash, deceased.

The genetic localization of the investigated genes, the number of exons, the genotyping markers and the families which were analyzed are summarized in Table 1. The pedigrees of all families analyzed in this study are shown in Figure 2. Primers used for amplification and sequencing are listed in Table 2.

**Table 1:** Investigated candidate genes and analyzed families.

Gene	Chromosomal location	Exons	Markers used for genotyping	Linkage-positive CVID families for the respective locus	Autosomal recessive CVID families	# of sporadic CVID patients
BCMA/*TNFRSF17*	16p13.1 11.9 Mb–12.0 Mb	3	D16S423, D16S407, D16S405	cv22, cv72, cv73, cv91, cv128, cv135, cv136	fr6, fr16, fr17, fr19, fr20, fr21, fr22, fr23, fr24, cv32	50
APRIL/*TNFSF13*	17p13.1 7.4 Mb	6	D17S513, D17S786	cv73, cv97, cv137	fr6, fr17, fr18, fr20, fr21, fr22, fr23, fr24, fr27	NA
*IL10*	1q31-q32 205 Mb	5	D1S1660, D1S1678, D1S1663, D1S2141, D1S229	cv73, cv77, cv125, cv128, cv136	fr6, fr16–fr26, fr28	5 (IBD)
*IL10RA*	11q23.3 117.3 Mb–117.4 Mb	7	D11S927, D11S925, D11S934	cv18, cv53, cv72, cv75, cv136	fr6, fr16–fr23, fr25, fr26, cv32	5 (IBD)
*IL10RB*	21q22.11 33.5 Mb–33.6 Mb	7	D21S265, D21S65, D21S219, D21S270, D21S167	cv4, cv22, cv32, cv75, cv80, cv128	fr6, fr16, fr17, fr18	4 (IBD)
*IL21*	4q26-q27 123.7 Mb–123.8 Mb	5	D4S430, D4S427	cv4, cv52, cv53, cv59, cv74, cv135	fr6, fr16–fr26, fr28–fr33, cv32	NA
*IL21R*	16p11 27.3 Mb–27.4 Mb	9	D16S420, D16S261, D16S411	cv4, cv22, cv72, cv77, cv137	fr6, fr16–fr26, fr28–fr33, cv32	NA
*CCL18*	17q11.2 31.4 Mb	3	D17S798, D17S250	cv18, cv78, cv80, cv93, cv125, cv136	fr6, fr16–fr26, fr28	NA

IBD, sporadic CVID patients with symptoms of inflammatory bowel disease; NA, not applicable.

**Table 2:** Primer sequences for candidate genes.

**Gene**	**Exon**	**Forward primer**	**Reverse primer**	**bp**^§^	**°C**^§^
*APRIL*	1	CCTTGCTACCCCACTCTTGA	TCTCTTGCACCCCCTTGAA	402	62
	2	CTGGGAAAAGGTGCGTGAGA	TGAGGAGAGAAGGAGGGAATC	337	62
	3	AGCAGCGTGGGGATTGTAA	CTCAGGTGCTTTTTGGTTCTTT	268	62
	4	AAGTGGATGCGGCTGAGATT	GGGAAGGGAGATGTTGAAGAA	536	62
	5	CATACCAAACCCAGCAGAC	GTTTCCAACCTCCCTCCTA	335	62
	6	GCGGGTCTGAGGAGTGAAGT	AGTCTAGGGGGTGGGAATGA	415	62
*BCMA*	1	GTCTATCTCCCTGGCACCTCTCAC	CACTGCCTTGCTTCCTTTCTCTTT	685	55
	2	CGGGAGGCTGAGGCAGGAG	TCGAAAGGGCTGTAACGAAGTGAA	451	55
	3	TCCCGACTGCTCTGTAGGCTAACG	TTCTTTCCCCCTCCCACCTTTCTC	797	57
*CCL18*	1	ATCCCTGGGTGCTTCCAACTC	TGCCCAGGGAGCCATCAA	414	57
	2	CCAAGCGGTGATATCTCCCAGTTC	AGGCCTTCCCCAAATGTCTCAGA	477	60
	3a	GAGGCCCCTGCAGTGTTTTGTG	CTGGGCATAGCAGATGGGACTCTT	498	60
	3b	GCAGGGGCCACAGGATTCC	TGCCCCTTTTCATATTTCCCTACT	680	57
*IL10*	1	GTGCCGGGAAACCTTGATTGTGG	GGGGGAATAGGTGTTGGGGATGG	435	60
	2 and 3	GGGCATCAAAAAGACCGCATTTCA	CCCCCAACGCCTGCTCAAAGA	763	60
	4	CCACCAGCTTGTCCCCTAAGTGTG	TGGCCGGCCAGCCTAACC	404	59
	5	AGCATGAGGGAGGGGAGCCTATTT	GCGCCCGGCCTAGAACCAA	781	57
*IL10RA*	1	CGCTAGCGCCCCAGGACAG	GTTTGCGGGTTGGGGGTTTG	545	64
	2	ATGTGCCCACTCTGCCCCTTACG	GGGCCCTCAGGCACTCACTTCATT	377	61
	3	GGGCTGTCCCAGTTTCTCCCAATG	CCCAACCCACCCCAAGACCTCTC	542	61
	4	CAAAGTCTCGGCGGGGACACC	CCTCCTCTGCCCACCCACCAT	388	61
	5	AGGCCCACCAGCTCTCAGTGTCC	CCAGGTGCACGCGTTTTGGATT	302	60
	6	CTTGGGCCACTCACTGAATGGTT	CCCACAGCGCTTGATGAAGGTAT	404	60
	7	CCTTCCCCGGCAGCACTGG	GCAGAGGAGCAGGCATGGCTAAAA	1048	63
*IL10RB*	1	GCGCCTTTCAAAGCTTGCGAGGAT	GGCCGTGGCGTTTGCATCTTCTCT	442	65
	2	ACCCACGTGGCCTTTGAAGACAT	GTGGCCACGAGAATTTCCCAGAC	416	60
	3	CCAGTCAGCCTCAGGGAGAC	GGGCCCCAACATAGTCACAT	486	54
	4	ACTTCCGTGGACTAATTGTTCTGC	CTTCAGGGAGGGAAAGGTCTG	494	55
	5	AGTCCCCCAAAGTGCTGTGATTAC	GGGCGATAGATTTCTGAACTGAGC	575	57
	6	TCTGAGACGTCCCCCAAGATAAAC	TGGGCAGCCCTAACTAGAAAGGAA	359	57
	7	CCAGCCAGGAGTTCTGTGAAAA	TAGATGTGGGGCTGGCTCAGAT	382	57
*IL21*	1 and 2	AGTTACTCACATTCATCCAT	CTGTCTCAGCAATACTACTT	736	50
	3	GAAATAGAGGATTGGGAAAG	GGTAAGGAAGACACCAGCAG	345	50
	4 and 5	TACAAGGACTTTTTCCATTG	GACTTTGCACACTTATGAGT	510	50
*IL21R*	1	TCTCTGCTGAGTGACCGTAAG	TTCCTTCCCCAGCCCGCTACA	675	58
	2	CAGCAAGGGCAAAATAGTCAG	TGTCTACCGCTTCTGTTTCAT	898	57
	3	TTGGGACATTTTCAGCATAGC	ACCATTCATGGGAAGCGTGTA	863	59
	4	TGCTGCCCTAAATGAGGTAGT	AAAATTAGCTGGGTGTAGTGG	610	59
	5	AGATGGGGTTTCACCGTGTTA	TGCTCGTGCCCTTGGTCTCTG	699	55
	6	TTGCTAAGATGTCTGTAGTTA	GGGAAATGGGTCTGGGGTAGA	1067	55
	7	CCCTGTTTTTCAGACGAGATA	TTCAGAGGTGTGGACTATTAC	755	55
	8	GTGAAGAGGTGGCTGTAATAG	AGATGGGGAGGCGGAGTGGTG	768	55
	9	AGTGAACCGAGATGGCACCAC	AGGGAGGACGGCTGTTGTCAT	1270	59

IBD, sporadic CVID patients with symptoms of inflammatory bowel disease; NA, not applicable.

Table 3 summarizes the observed sequence variations, the *in silico *analysis of missense mutations by SIFT [29] and Polyphen [30] and the genotype frequencies in the studied CVID cohort compared to healthy donors.

**Table 3:** Description and analysis of SNPs found in CVID patients

Gene	SNP	SIFT^[30]^	Polyphen^[31]^	CVID Genotypes			Healthy donors Genotypes			Additional studies
*BCMA*	S81N	tolerated	benign	T/T	T/C	C/C	T/T	T/C	C/C	n.a.
	rs373496			0	0,014	0,986	0*	0,050*	0,950*	
	synonymous (T159)	n.a.	n.a.	T/T	T/C	C/C	T/T	T/C	C/C	no aberrant
	rs2017662			0,071	0,029	0,900	0*	0,136*	0,864*	splicing of mRNA
	synonymous (T175)	n.a.	n.a.	T/T	T/C	C/C	T/T	T/C	C/C	no aberrant
	rs2071336			0,071	0,029	0,900	0*	0,117*	0,883*	splicing of mRNA
	K179Q	**not tolerated**	benign	C/C	C/A	A/A	C/C	C/A	A/A	no segregation, normal expression
				0	0,014	0,986	n.k.	n.k.	n.k.	
*APRIL*	G67R	tolerated	benign	A/A	A/G	G/G	A/A	A/G	G/G	n.a.
	rs11552708			0	0,190	0,810	0,033*	0,233*	0,733*	
	N96S	tolerated	benign	G/G	G/A	A/A	G/G	G/A	A/A	n.a.
	rs3803800			0,632	0,316	0,053	0,683*	0,183*	0,133*	
*IL10*	3' UTR	n.a.	n.a.	C/C	C/T	T/T	C/C	C/T	T/T	n.a.
	rs3024496			0,210	0,368	0,421	0,293*	0,500*	0,207*	
*IL10RA*	synonymous (A153)	n.a.	n.a.	A/A	A/G	G/G	A/A	A/G	G/G	n.a.
	rs2256111			0,154	0,346	0,500	0,328*	0,448*	0,224*	
	I224V	tolerated	benign	A/A	A/G	G/G	A/A	A/G	G/G	n.a.
	rs17121493			0,846	0,154	0	0,917*	0,083*	0*	
*IL21*	synonymous (C78)	n.a.	n.a.	G/G	G/A	A/A	G/G	G/A	A/A	n.a.
	rs4833837			0,200	0,360	0,440	0,174**	0,522**	0,304**	
*IL21R*	5' UTR rs961914	n.a.	n.a.	T/T	T/C	C/C	Allele T	Allele C		n.a.
				0,042	0,167	0,792	0,140***	0,860***		
	T46M	tolerated	benign	T/T	T/C	C/C	T/T	T/C	C/C	n.a.
				0	0,042	0,958	0	0,010	0,990	
	R275Q	tolerated	benign	A/A	A/G	G/G	A/A	A/G	G/G	n.a.
				0	0,042	0,958	0	0,050	0,950	

The observed known and newly discovered SNPs are listed in the table. The table summarizes in silico analysis results by SIFT and Polyphen for missense mutations and the frequencies of SNPs in CVID patients versus healthy control populations. n.a., not applicable. n.k., not known. * HapMap CEU, ** PGA-EUROPEAN-PANEL, *** CEPH

### BCMA/*TNFRSF17 *and APRIL/*TNFSF13*

Seventeen probands of CVID families were analyzed. Seven of these families were selected due to being linkage-positive at the *TNFRSF17 *locus on chromosome 16p (Table 1). In addition, we expanded our mutation screening in BCMA/*TNFRSF17 *to a larger cohort of 50 sporadic CVID cases. One previously described heterozygous single nucleotide polymorphism (SNP) was found in a proband of family cv22 in exon 2: Ser81Asp [dbSNP: rs373496]. In exon 3 two synonymous SNPs [dbSNP: rs2017662 and rs2071336] were found in homozygous state in five individuals (family fr24 and four sporadic cases) and in heterozygous state in probands of families cv73 and fr21. RT-PCR was performed but evidenced no alternative splicing products (data not shown). A novel variant was identified in exon 3: K179Q, which was present in one individual. The K179Q variant was present in the heterozygous state both in affected and healthy members of the family, which renders unlikely a contribution of this mutation to the pathogenesis of the hypogammaglobulinemia. Flow cytometric analysis confirmed normal expression of the protein (data not shown).

APRIL/*TNFSF13 *was analyzed in the probands from 12 CVID families. The families cv73, cv97 and cv137 were linkage positive at the *TNFSF13 *locus (Table 1). Two previously described non-synonymous SNPs [dbSNP: rs11552708 and rs3803800], located in exon 1 and exon 2 respectively, were found in the studied cohort (Table 3).

### IL10, IL10 receptor α and IL10 receptor β

The *IL10 *gene was sequenced in 23 individuals. Of those, 13 represented autosomal recessive CVID families, five families were selected for being linkage-positive, and five were sporadic CVID cases showing a clinical phenotype compatible with inflammatory bowel disease (Table 1). One previously described heterozygous SNP [rs3024496] located in the 3'UTR of the *IL10 *gene was found in frequencies comparable to that in healthy controls (Table 3).

IL10 receptor α, encoded by *IL10RA *was analyzed in 22 individuals, of whom 12 belonged to autosomal recessive families, five were from linkage-positive families at the *IL10RA *locus, and five were sporadic CVID cases (Table 1). The known synonymous SNP [dbSNP: rs2256111] in exon 4 and the SNP [dbSNP: rs17121493] in exon 5 (I224V) were observed in frequencies comparable to those which are reported for normal controls in public databases (Table 3).

The *IL10RB *gene was analyzed in 14 CVID patients, 10 from CVID families (six of them linkage-positive at the *IL10RB *locus) and four sporadic CVID cases (Table 1). We found no genetic alterations in the 7 exons of *IL10RB*.

### IL21 and IL21 receptor

The 5 exons of *IL21 *were amplified and sequenced in 25 individuals (19 autosomal recessive CVID (AR-CVID) families and 6 autosomal dominant CVID families (AD CVID) that are linkage-positive at the *IL21 *locus). In exon 3 of the *IL21 *gene the synonymous change [dbSNP: rs4833837] was observed in a frequency comparable to that reported in public databases (Table 3).

**Figure 3:**
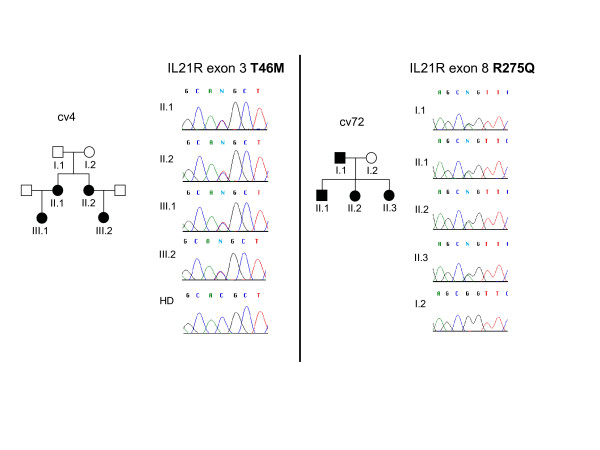
**Two new polymorphisms in the *IL21R *gene**. Left panel: pedigree of CVID family cv4 and sequence chromatogram of *IL21R *exon 5 showing a C to T transition (c.137 C > T), resulting in T46M at the protein level; right panel: pedigree of CVID family cv72 and sequence chromatogram of *IL21R *exon 8 showing a G to A transition (c.824 G > A), resulting in R275Q at the protein level; symbols: circles, female; squares, male; filled symbols, affected individual.

The *IL21R *gene was analyzed in 24 individuals (19 AR CVID families and 5 AD CVID families linkage-positive at the *IL21R *locus). In exon 1 of *IL21R *the SNP [dbSNP: rs961914] was observed in homozygous state in the proband from family fr18 and in heterozygous state in cv32, fr25, fr28 and fr29. In exon 5 of the *IL21R *gene we found a previously undescribed heterozygous SNP resulting in the replacement of a conserved threonine by methionine at position 46 in the proband from family cv4 (Figure 3). In addition, a new heterozygous change in exon 8 resulting in R275Q was found in the family cv72. Both heterozygous substitutions showed perfect segregation with the disease phenotype in these families (Figure 3). However, we subsequently screened a cohort of 100 healthy individuals with normal immunoglobulin levels and found respectively 2 (T46M) and 5 (R275Q) heterozygous individuals among them, suggesting that these two coding sequence changes may not be disease-associated.

### CCL18

The three exons of *CCL18 *including the 5'and 3'untranslated region were amplified and analyzed in 19 probands including six from CVID families linkage-positive at the *CCL18 *locus on chromosome 17 (Table 1). No genetic alterations were found.

## Discussion

In recent years, genetic linkage analysis of selective IgA deficiency (sIgAD) and common variable immunodeficiency (CVID) has led to the identification of several susceptibility loci [10-12]. However, all four molecular genetic defects underlying CVID reported to date, namely ICOS [13], TACI [15,16], CD19 [17] and BAFF-R [31] were identified by a candidate gene approach using either phenotypic or genetic screening of genes known to be involved in B cell homeostasis, B cell activation or the T- B cell interaction. In the case of TACI/*TNFRSF13B*, linkage analysis was used to identify one small CVID family with the same homozygous mutation in two individuals [15].

The success with four CVID candidate genes stimulated researchers to evaluate additional candidate genes that encode proteins in the pathways of ICOS, TACI, BAFF-R, and CD19 signaling [32,33]. Here we report on the analysis of eight additional candidate genes in CVID, where no mutation was identified.

The candidacy of the genes encoding BCMA and APRIL was strong since their close relatives BAFF-R and TACI have recently been shown to be mutated in CVID [15,16,31]. Although both *TNFRSF17 *and *TNFSF13 *were shown to be polymorphic in the studied cohort, none of these changes were obviously disease related.

The genes encoding IL10 and its receptor subunits were selected as candidate genes for CVID, since we were able to show that IL10 is the critical cytokine missing in ICOS deficiency [16]. Furthermore, IL10 plays an important role in B cell development. However, no mutations were found in the investigated families.

In a context dependent manner IL21 is able to induce differentiation of plasma cells and memory cells, or is able to promote apoptosis of resting and anti-IgM-activated B cells [25]. Ettinger et al recently showed that IL21 costimulation in humans is capable of inducing substantial B-cell expansion, plasma cell differentiation from CD27+ memory B-cells and class switch recombination [27].

Therefore, we screened multiple families for mutations in *IL21 *and/or *IL21R*. Apart from known SNPs we could identify two new heterozygous variations in IL21R that result in amino acid changes and segregated with the disease phenotype in families. However these changes were also found in a screening of healthy individuals. This suggests that these two variations may not be disease associated.

## Conclusion

In summary, we cannot provide evidence that the above listed eight genes are implicated in the etiology of CVID in this studied cohort of patients. However, only protein coding regions of the respective genes were analyzed leaving the possibility of mutations in promoter or regulatory regions. Based on other studies of humans and mice these genes remain attractive candidate genes for CVID. Larger CVID cohorts or cohorts of non-European origin may enable the identification of rare mutations in these genes.

## Methods

### Notation convention used throughout this paper

For several of the genes we mention, such as TACI/*TNFRSF13B*, the HUGO-approved gene acronym (*TNFRSF13B*) has no resemblance to the protein acronym (TACI), which is much more familiar to immunologists. In those cases, we use either the protein name alone or the compound notation protein/*gene*, when referring to either the gene or the protein it encodes.

### Patients and families

Twenty families with at least one case of CVID were selected from a collection of 101 multiplex CVID/IgA deficient families [5,9,10,34], based in part on reanalysis of genotype data previously generated for genetic linkage studies. This method had successfully identified family A as consistent with linkage to the TACI/*TNFRSF13B *locus [15]. In addition, 19 autosomal recessive CVID families were collected at the Department of Rheumatology and Clinical Immunology in Freiburg, Germany, from 2001 to 2005. The families were considered as having possible recessive inheritance when two or more siblings in one generation were affected by CVID and their parents and children had normal immunoglobulin levels. Alternatively, ten families were considered as recessive because of a known consanguinity in these families. Six families originated from Turkey, five from Germany, four from Italy, two from the UK and one from Sweden. For the analysis of *TNFRSF17*, encoding BCMA, fifty additional individuals with sporadic CVID followed in the Department of Pediatrics, University of Brescia were included in this study. Informed written consent was obtained from each patient or parent guardians prior to participation under the internal ethics review board-approved clinical study protocol (ZERM, University Hospital Freiburg (#239/99).

### Linkage analysis

Genetic linkage analysis was carried out by computing LOD scores using a dominant model and imperfect penetrance using FASTLINK [35-37], as previously described [11]. We also tried a recessive model in which the penetrance for heterozygotes is assumed to be the same (0.05) as for individuals with no disease-associated alleles. For each candidate gene, we tried to find close flanking markers from the marker set chosen for the previous studies [9,10]. The relevant markers near each gene are shown in Table 1. We tried to find families with positive scores at all flanking markers, but sometimes had to settle for families with positive scores at one or some flanking marker(s) and 0 scores (either due to no genotypes or an uninformative marker) at some flanking marker(s). With the exceptions of cv32 and cv74, all the families are small and have maximum achievable scores under 1.0. Thus, the positive scores should be taken only as evidence that these families are consistent with linkage and preferred over families with negative LOD scores, but we make no claims of statistically significant linkage. Based on these criteria, the families which were consistent with linkage for each individual candidate gene were selected out of the previously described cohort [9,10] (Table 1, Figure 1).

### Sequencing of candidate genes

Candidate genes were evaluated by sequencing the coding regions of the genes on genomic DNA including 20 bp of the flanking intronic or untranslated regions. All primers were sought with the aid of the Primer Select software (PE Applied Biosystems, Foster City, CA, USA); sequences are summarized in Table 2. Genomic DNA was amplified by PCR and subsequently sequenced with the amplification primers. After gel electrophoresis on an ABI Prism™ 377 DNA Sequencer, the data was analyzed by the DNA Sequencing Analysis software, version 3.4 (PE Applied Biosystems) and Sequencer™, version 3.4.1 (Gene Codes Corporation, Ann Arbor, MI, USA).

## Authors' contributions

US evaluated candidate genes and wrote the manuscript. CN, JT, CW, QP, VL, TH, JJ, JB, LD performed sequencing of candidate genes, evaluated primary data and participated in preparation of the manuscript. AM, AP, VM collected and evaluated CVID families and provided DNA samples for candidate gene analysis. AW, LH collected and evaluated CVID families for linkage analysis and provided DNA from these families. AS evaluated genetic linkage analysis data and selected families for analysis of candidate genes. BG designed and supervised the study and wrote the manuscript. All authors have read and approved the final version of the manuscript.
